# Genomic divergence between nine- and three-spined sticklebacks

**DOI:** 10.1186/1471-2164-14-756

**Published:** 2013-11-05

**Authors:** Baocheng Guo, Frédéric JJ Chain, Erich Bornberg-Bauer, Erica H Leder, Juha Merilä

**Affiliations:** Ecological Genetics Research Unit, Department of Biosciences, University of Helsinki, Helsinki, Finland; Department of Evolutionary Ecology, Max Planck Institute for Evolutionary Biology, Plön, Germany; Institute for Evolution and Biodiversity, Evolutionary Bioinformatics, Westfalian Wilhelms University of Münster, Münster, Germany; Division of Genetics and Physiology, Department of Biology, University of Turku, Turku, Finland

**Keywords:** *Pungitus pungitius*, *Gasterosteus aculeatus*, Comparative genomics, Transcriptome, Substitution rate, Adaptive evolution

## Abstract

**Background:**

Comparative genomics approaches help to shed light on evolutionary processes that shape differentiation between lineages. The nine-spined stickleback (*Pungitius pungitius*) is a closely related species of the ecological ‘supermodel’ three-spined stickleback (*Gasterosteus aculeatus*). It is an emerging model system for evolutionary biology research but has garnered less attention and lacks extensive genomic resources. To expand on these resources and aid the study of sticklebacks in a phylogenetic framework, we characterized nine-spined stickleback transcriptomes from brain and liver using deep sequencing.

**Results:**

We obtained nearly eight thousand assembled transcripts, of which 3,091 were assigned as putative one-to-one orthologs to genes found in the three-spined stickleback. These sequences were used for evaluating overall differentiation and substitution rates between nine- and three-spined sticklebacks, and to identify genes that are putatively evolving under positive selection. The synonymous substitution rate was estimated to be 7.1 × 10^-9^ per site per year between the two species, and a total of 165 genes showed patterns of adaptive evolution in one or both species. A few nine-spined stickleback contigs lacked an obvious ortholog in three-spined sticklebacks but were found to match genes in other fish species, suggesting several gene losses within 13 million years since the divergence of the two stickleback species. We identified 47 SNPs in 25 different genes that differentiate pond and marine ecotypes. We also identified 468 microsatellites that could be further developed as genetic markers in nine-spined sticklebacks.

**Conclusion:**

With deep sequencing of nine-spined stickleback cDNA libraries, our study provides a significant increase in the number of gene sequences and microsatellite markers for this species, and identifies a number of genes showing patterns of adaptive evolution between nine- and three-spined sticklebacks. We also report several candidate genes that might be involved in differential adaptation between marine and freshwater nine-spined sticklebacks. This study provides a valuable resource for future studies aiming to identify candidate genes underlying ecological adaptation in this and other stickleback species.

**Electronic supplementary material:**

The online version of this article (doi:10.1186/1471-2164-14-756) contains supplementary material, which is available to authorized users.

## Background

The rapid advances in sequencing technologies have facilitated the development of comparative genomics – an important approach in contemporary evolutionary biology research [1, 2]. The stickleback fishes (Gasterosteidae) provide an excellent model system for such comparative studies. The three-spined stickleback (*Gasterosteus aculeatus*) has become a vertebrate ‘supermodel’ allowing a combination of studies at molecular, developmental, phenotypic, and population genetic levels to explore factors and processes relevant for adaptive evolution in ecologically relevant contexts [3, 4]. The three-spined stickleback is a small teleost populating diverse ecosystems across a wide geographic distribution in the northern hemisphere and occurs in marine, brackish, and freshwater habitats. Populations that have colonized freshwater habitats after the retreat of Pleistocene ice sheets have evolved remarkable morphological and behavioral diversity as compared to marine populations [5, 6]. For example, they have repeatedly evolved changes in body shape, skeletal armor, trophic apparati, pigmentation, osmoregulatory functions, life history, and behavior [5]. The genetic architecture for several of these phenotypic adaptations has been – or is being – deciphered [7–15]. Interestingly, the parallel evolution (similar phenotypes evolving independently in different populations derived from a common ancestor) of armor loss, pelvic reduction, and pigmentation has been found to result from parallel genetic changes in similar genes [8, 9, 11, 14]. However, relatively little is known about the genetics of these or other traits in other stickleback species (but see: [16–20]).

The nine-spined stickleback (*Pungitius pungitius*) is an emerging model for evolutionary biology research [21] and has diverged from the three-spined stickleback around 13 million years ago (Mya) [22], but the two species are ecologically – and to some degree also phenotypically – very similar [23]. Phylogeographic and population genetic analyses of the nine-spined stickleback demonstrate that their colonization and adaptation to freshwater habitats from marine environments has occurred independently multiple times [24–26]. Meanwhile, freshwater nine-spined sticklebacks have also evolved – repeatedly and independently – similar morphological [26–28], behavioral [29, 30], neurological [31–36], and physiological [37, 38] phenotypes in different localities. Notably, similar adaptive traits also have been evolved in parallel between nine- and three-spined sticklebacks [6]. For example, both marine nine- and three-spined sticklebacks have a complete pelvis, but several different freshwater populations in both species have undergone a genetically based reduction - or even total loss - of the pelvic girdle and associated spines [16–18]. However, it is still uncertain whether or not the genetic underpinnings of the pelvic reduction in nine- and three-spined sticklebacks are the same. For instance, Shapiro et al. [16] first suggested that changes of *Pitx1* expression might contribute to pelvic reduction in both species, but later discovered that the major loci controlling for pelvic development were completely different between the two species. This suggests that the pelvic reduction in these species is an example of genetic convergence [17] (but see [18]). Hence, nine- and three-spined sticklebacks offer a powerful opportunity to study whether or not similar phenotypic changes across species are associated with the same genes or genetic mechanisms.

A genome-wide comparative study can help us to better understand how selection has shaped divergence and illuminate the genetic basis of parallel evolution in nine- and three-spined sticklebacks. It can also reveal the extent of genome-wide differentiation across protein-coding and non-coding regions and the prevalence of species-specific genes that may influence the evolutionary trajectory of divergent species. However, compared to the three-spined stickleback with abundant genomic resources [3, 39, 40], genomic resources for the nine-spined stickleback are still largely lacking (but see: [17, 41, 42]). For example, development of microsatellite markers for study of nine-spined stickleback currently is based on the three-spined genome, but cross-species utility of microsatellite primers is limited due to low amplification success [43]. Fortunately, the recent explosion of affordable Next Generation Sequencing (NGS) technology provides evolutionary and ecological researchers a great opportunity to conduct genome-wide studies of non-model organisms with limited genetic and genomic resources [44–46]. For instance, transcriptome, a collection of expressed sequences, represents a sample of the spatiotemporally expressed genome that can be used for comparative genomic studies at an interspecific level, as well as genetic diversity analyses at an intraspecific level. Here, we used deep sequencing (454 GS FLX) to characterize partial brain and liver transcriptomic libraries of nine-spined sticklebacks from marine and freshwater populations exhibiting a high degree of morphological and genetic divergence [26–38, 41]. With the resulting transcripts, we (1) characterized the sequence divergence between the two closely related stickleback species, (2) investigated rates of molecular evolution for patterns consistent with positive selection, and (3) evaluated sequence differentiation between marine and freshwater nine-spined sticklebacks.

## Results

### Sequencing and assembly

We obtained a total 337,630 high quality reads with mean length of 250 bp from 454 sequencing of four cDNA libraries from nine-spined sticklebacks (Additional file 1: Figure S1). Contig assembly of the reads were combined from the four cDNA libraries into one “nine-spined stickleback transcriptome” containing 7,932 contigs ≥ 100 bp (median = 403 bp, Additional file 1: Figure S2) with an average coverage depth of 38 reads (Additional file 2: Table S1).

### Functional annotation

A BLASTX search returned 3,347 (42.2%) nine-spined stickleback contigs with significant hits to three-spined stickleback genes. This proportion of contigs with BLAST hits is similar to previous transcriptome studies [47–49], in which contigs without significant hits may consist of untranslated transcripts, chimeras or assembly artifacts. Blast2Go with the Gene Ontology (GO) annotations database was used for further annotation and 2,071 contigs have one or more GO terms (Additional file 1: Figure S3). We additionally found that 104 contigs had no significant BLASTX hit with protein sequences from the three-spined stickleback but had significant hits with protein sequences in at least one of the other seven fish genomes available from Ensembl. By using BLASTN and BLAT searches, we confirmed that 15 of the 104 contigs had no hits in the current three-spined stickleback genome (Additional file 2: Table S2). Because these contigs correspond to genes in other teleost genomes, this suggests that the orthologous sequences of these contigs have probably been lost in the three-spined stickleback rather than gained in nine-spined sticklebacks.

### Sequence comparison between nine- and three-spined sticklebacks

**Figure 1:**
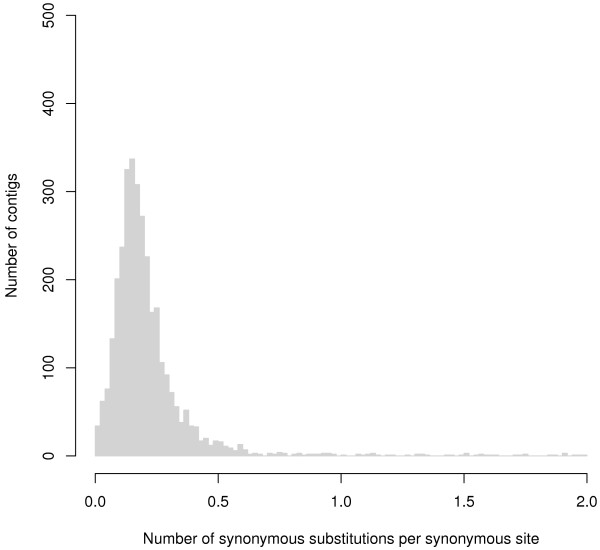
**Distribution of** ***K***_***s***_ **distances between nine-spined stickleback contigs and their three-spined stickleback orthologs.**

We found that 3,091 out of the 3,347 nine-spined stickleback contigs (92.4%) had a pairwise *K*_*s*_ ≤ 0.5 compared to their three-spined stickleback orthologs (Figure 1), and these had an average length of 690 bp (Additional file 1: Figure S4). We restricted all further analyses to these 3,091 contigs, or “unigenes”, in an attempt to curtail the effects of erroneously called orthologs with large *K*_*s*_ values. The corresponding genes are more or less evenly distributed across the three-spined stickleback genome with 2.3% to 7.1% of genes on each chromosome, and the gene number per chromosome is positively correlated with chromosome size (*r*_*s*_ = 0.84, *P* < 1.2 × 10^-6^, in Additional file 1: Figure S5). Given the conserved genomic synteny between the two species [35], these observations suggest that the unigenes are a relatively unbiased sample of nine-spined stickleback genes in terms of genomic distribution.

**Figure 2:**
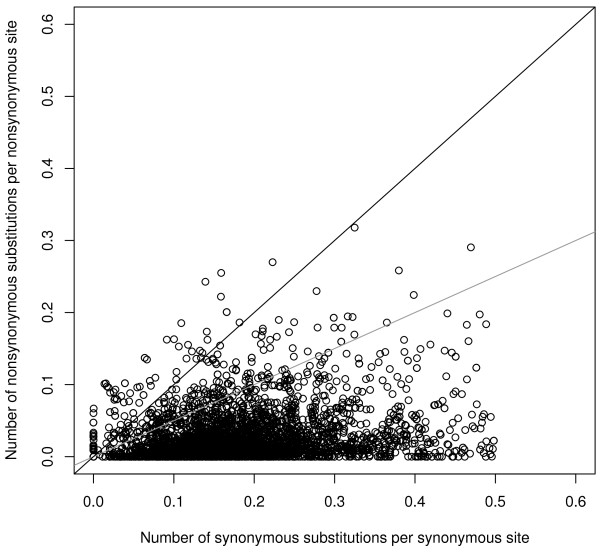
**Distribution of pairwise** ***K***_***a***_ **and** ***K***_***s***_ **distances between nine-spined stickleback unigenes and their three-spined stickleback orthologs.** Genes with *K*_*a*_/*K*_*s*_ ratio >1 fall above the black line while those with *K*_*a*_/*K*_*s*_ ratio between 0.5 and 1 fall between the gray and black lines.

We used three methods to detect positive selection on genes in sticklebacks. We first calculated the pairwise substitution rates *K*_*s*_, *K*_*a*_, and *K*_*a*_/*K*_*s*_ between the nine-spined stickleback unigenes and their putative orthologs in the three-spined stickleback (Figure 2). Genes are generally under strong purifying selection (low *K*_*a*_/*K*_*s*_ values), with a mean pairwise *K*_*s*_ value was 0.1841 ± 0.0017 (mean ± SD). A total of 194 (6.3%) orthologous pairs had a *K*_*a*_/*K*_*s*_ ratio between 0.5 and 1 (points above the grey line in Figure 2), and 74 (2.4%) had a *K*_*a*_/*K*_*s*_ ratio > 1 (points above the black line in Figure 2). The latter 74 unigenes are distributed across 16 chromosomes (Additional file 3: Table S3).

We also performed the branch-site test with medaka as an outgroup to detect positive selection operating on sites along each stickleback lineage. The branch-site test revealed a total of 33 unigenes (*p* < 0.05, eight after multiple test correction with q-value < 0.05) that are putatively evolving under positive selection in the nine-spined stickleback lineage and 39 unigenes (seven after multiple test correction) in the three-spined stickleback lineage (Additional file 3: Table S4). We also found 82 unigenes (37 after multiple test correction) with sites evolving under positive selection in the ancestral lineage before the split between nine- and three-spined sticklebacks (Additional file 3: Table S4).

A third method was used for inferring positive selection by utilizing nine-spined stickleback SNPs. We analyzed the patterns of selection among genes with the MK test and the direction of selection (DoS). We found 48 unigenes that departed from neutrality (Chi-square test with Yates correction, df = 1, *p* < 0.05), 18 of which show a signature of positive selection (Additional file 3: Table S5). However, none of these signatures remained statistically significant after correction for multiple tests.

**Figure 3:**
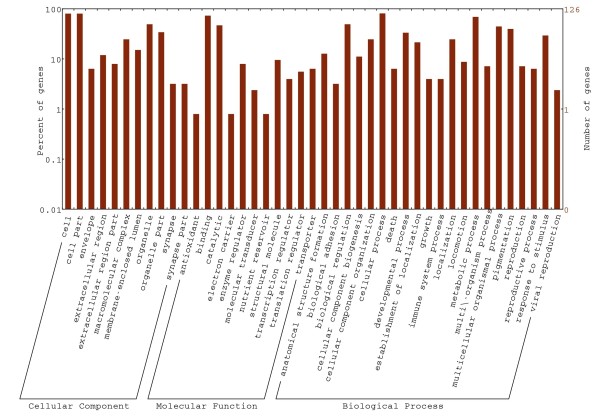
**GO assignment for genes showing adaptive evolution between nine- and three-spined sticklebacks.** GO annotation were retrieved using Blast2GO followed by classification and plotting with WEGO [93].

It is noteworthy that positive selection on seven genes was detected by at least two of the three methods mentioned above. For example, two genes with a pairwise *K*_*a*_/*K*_*s*_ ratio ≥ 1 that are involved in lipid transport are also detected using the branch-site test, of which one gene (apolipoprotein B - ENSGACG00000009637) is consistent with positive selection in the nine-spined stickleback lineage and the other gene (a vitellogenin gene - ENSGACG00000009711) is consistent with positive selection in the three-spined stickleback lineage. Other overlaps from methods of detecting positive selection include a gene (adenylate cyclase 6 - ENSGACG00000008575) detected by the MK test and the branch-site test in the nine-spined stickleback lineage, and four genes (complement factor H-related 3 - ENSGACG00000001733, fetuin B - ENSGACG00000005690, HECT domain containing E3 ubiquitin protein ligase 1 - ENSGACG00000012853 and an uncharacterized gene - ENSGACG00000007507) detected by both pairwise *K*_*a*_/*K*_*s*_ and the MK test. Combining all three tests, we found a total of 165 genes with patterns of adaptive evolution in either the nine- or three-spined stickleback, or both. These genes are distributed rather evenly across all of the three-spined stickleback chromosomes except XIV (Additional file 1: Figure S5). We found 126 of these 176 genes with associated GO annotations spanning a broad range of functions (Figure 3). We found that nine GO terms (*viz.* translational elongation, macromolecule biosynthesis, protein biosynthesis, translation, cellular biosynthesis, physiological process, macromolecule metabolism, biosynthesis, and energy derivation by oxidation of organic compounds) were significantly overrepresented among these 165 genes by comparing to all three-spined stickleback genes, which suggested that these 165 genes have been subject to adaptive evolution (family-wise error rate, *P* < 0.05; Additional file 3: Table S6).

A total of 368 nine-spined stickleback unigenes contained partially sequenced UTRs ≥ 50 bp. The average K2P distance of these UTRs and their three-spined stickleback orthologous sequence was 0.0709 ± 0.0020 (median = 0.0688), whereas the average K2P distance of the coding regions for these same genes was 0.0513 ± 0.0017 (median = 0.0436). The average pairwise *K*_*s*_ for the 368 unigenes was 0.1746 ± 0.0047 (median = 0.1637) and is close to that of the all 3,091 unigenes (mean = 0.1841 ± 0.0017; median = 0.1687), which suggests no bias of the 368 unigenes with UTR information, at least with respect to *K*_*s*_. The divergence of UTRs was significantly higher as compared to the divergence in corresponding coding regions (Wilcoxon signed rank test, *P* < 2.2 × 10^-16^) but significantly lower than that of *K*_*s*_ (Wilcoxon signed rank test, *P* < 2.2 × 10^-16^), which suggests that UTRs have evolved under lower selective pressures than coding regions, albeit not neutrally (assuming that synonymous sites evolve close to neutrally). Based on the divergence estimates above and the species divergence time of 13 Mya [22], we calculated the substitution rate as 2.0 × 10^-9^ per site per year in coding regions (including both synonymous and nonsynonymous sites) and 2.7 × 10^-9^ per site per year in UTRs between nine- and three-spined sticklebacks.

### Divergence between marine and freshwater nine-spined sticklebacks

We found 1,814 SNPs (0.044% of evaluated genic sites) among 718 unigenes in the sampled nine-spined sticklebacks (934 unique SNPs in the marine sample, 642 unique SNPs in the pond sample, and 238 SNPs shared in common). Many of the SNPs (567) are predicted to be nonsynonymous changes, while the remaining are either synonymous (665) or in UTRs (582) (Additional file 3: Table S7). We found 47 SNPs in 28 unigenes (spanning 25 different genes) that lead to ‘fixed’ genotypes between the two ecotypes, including 17 homozygous differences. These divergent SNPs occur in both tissue types and as such are not tissue-specific differences but most probably reflect general genetic differences between the ecotypes (at least from the sampled individuals). Of the fixed homozygous differences, five are nonsynonymous SNPs, ten are synonymous SNPs and two are SNPs found in UTRs (Additional file 3: Table S8).

### Discovery of microsatellite markers

Microsatellites are important genetic markers for non-model organisms and have been widely used for studies of nine-spined sticklebacks [18, 19, 24, 41, 43]. We analyzed the nine-spined stickleback unigenes to identify microsatellite markers. We obtained 468 SSRs in 358 unigenes (Additional file 3: Table S9). In terms of abundance, dinucleotide repeats were most abundant (178, 38.0%) followed by trinucleotide repeats (148, 31.6%), mononucleotide repeats (139, 29.7%), 1 tetranucleotide repeat, and 2 hexanucleotide repeats. Of the 468 SSRs, 428 are perfect and 40 are compound. AC/GT (124 out of 178, 69.7%) was the most abundant dinucleotide repeat motif and AGG/CTT (58 out of 148, 39.2) was the most abundant trinucleotide repeat motif.

## Discussion

### The nine-spined stickleback transcriptome

In recent years, the use of comparative genomic approaches in a phylogenetic framework has shed much light on a variety of fundamental evolutionary questions, such as adaptive evolution [3, 50, 51], genetic variation [52–55], and speciation [56–58]. Development of genomic resources is the first step towards such biological questions. Using 454 pyrosequencing, we have contributed to the improvement of genomic resources for nine-spined sticklebacks. We provide over three thousand transcript sequences that correspond to an orthologous gene in the three-spined stickleback, and report hundreds of genic microsatellites that can be used as markers in future experiments with nine-spined sticklebacks. The data provided here significantly increase the number of available gene sequences for nine-spined sticklebacks since there are currently fewer than 1,000 sequence entries in the National Center for Biotechnology Information. Given its status as an emerging model for evolutionary biology research [21], this transcriptomic data will be of interest to researchers investigating the evolution of nine-spined sticklebacks, for example by using the identified SNPs or microsatellite markers for population genetics studies. It also allows for more refined inferences concerning stickleback and teleost evolution in a phylogenetic framework by providing orthologs of closely related fish species. Thus, apart from contributing a large number of new gene sequences to the research domain, the results of this study represent the first reported nine-spined stickleback transcriptomic resource, and as such, provide a starting point for intra- and inter-specific genomic comparisons in sticklebacks.

### Sequence divergence between nine- and three-spined sticklebacks

The nine-spined stickleback transcriptome characterized in this study allowed us to survey sequence divergence between two closely related species – nine- and three-spined sticklebacks. Because the two species diverged 13 Mya [22], we anticipated that the genetic differences would be considerable despite the highly ecological, phenotypic, and genetic similarities between the species [23, 43]. The rate of sequence substitution is of central importance to understand mechanisms underlying molecular evolution. Rates of nonsynonymous and synonymous substitutions are good indicators of selective pressures at the sequence level of protein-coding genes [59, 60]. Synonymous sites usually evolve neutrally and can provide insights on the background rate of sequence evolution [59, 60], thus we used the *K*_*s*_ values of protein-coding genes to estimate neutral substitution rates in sticklebacks. The average substitution rate was estimated to be 7.1 × 10^-9^ per synonymous site per year between nine- and three-spined sticklebacks (Additional file 1: Figure S6) when calibrated to the divergence time of 13 Mya. This rate is faster than previously published genome-wide substitution rate estimates available across mammals (2.2 × 10^-9^ per synonymous site per year; [61]), but is nearer the substitution rate of teleosts (1.25 × 10^-6^ in cichlids; [46]) as the rates of molecular evolution in fish are known to be fast compared to other vertebrates [62]. Additionally, the unigenes we identified may be enriched with highly expressed genes that are more easily detected in transcriptomic sequencing, and thus the estimated substitution rate might be an underestimation because highly expressed protein coding genes usually evolve slowly [63]. Nevertheless, this estimated substitution rate should be a useful yardstick for research in teleost molecular evolution in general, and particularly for those studies on stickleback phylogeny and molecular clock dating.

Identifying genes that show evidence of positive selection can help us in understanding whether closely related species occupying similar ecological niches share genetic attributes involved in adaptation. The *K*_*a*_/*K*_*s*_ ratio (= 1: neutral evolution; > 1: positive selection; < 1: purifying selection) is often used for diagnosing the extent and direction of selection on sequence evolution [59, 60]. Using three analyses based on nonsynonymous and synonymous substitutions, a total of 165 genes show indications of positive selection in one or both species of sticklebacks. These 165 genes have significantly smaller pairwise *K*_*s*_ (Wilcoxon–Mann–Whitney test, *P* = 1.4 × 10^-7^) but significantly larger pairwise *K*_*a*_ (Wilcoxon–Mann–Whitney test, *P* = 5.7 × 10^-4^) compared to the other analyzed genes (Additional file 1: Figure S7). Despite a broad range of GO annotations that these genes are involved with, we found that they showed enrichment in several functional categories. Such genes may be of particular interest for further studies aiming to investigate their detailed functions, as well as possible associations with ecological differences between stickleback species.

In addition to coding sequence changes, regulatory sequence changes may play an important role in repeated adaptive evolution of freshwater three-spined sticklebacks [3]. In general, UTRs, especially 3′-UTRs, are found to evolve neutrally among very closely related taxa [46]. However, we found that UTRs between nine- and three-spined sticklebacks are under stronger purifying selection as compared to synonymous sites, but under more relaxed selection as compared to coding regions (both synonymous and nonsynonymous sites). These findings suggest that some UTRs may be important in shaping stickleback evolution [3].

Gene gains and losses are important processes contributing to evolutionary innovation and differentiation [64, 65], perhaps especially so in teleosts because of the teleost-specific whole genome duplication event [66]. The comparison between stickleback orthologs revealed that some genes are likely to have been lost in the three-spined stickleback, as they exist both in nine-spined sticklebacks and other model fish genomes. It is also possible that these genes are missing from the current three-spined stickleback genome assembly, or that the genes have evolved so rapidly that they no longer resemble the same gene in other fishes. Of the genes that might have been lost in three-spined sticklebacks, nine have associated GO terms related to binding (protein and iron), cell migration, and membrane component. However, a more complete grasp of the number of genes differentially lost and retained between nine- and three-spined sticklebacks can only be answered with a complete nine-spined stickleback genome. Nevertheless, our results suggest that as in the case of other vertebrates [65, 67], stickleback divergence is also accompanied with gene losses.

However, we are aware that our results largely depend on the initial dataset for which we can make comparisons between genes. Because we used a subset of all genes in the genome, we cannot capture the entire list of variation and genes that are evolving under positive selection. In fact, our dataset may further be biased towards slowly evolving genes under stronger purifying selection if we are capturing mainly highly expressed genes, and those with low *K*_*s*_ values. Nevertheless, our results should provide a useful first step towards unraveling the genetics underlying divergence between nine- and three-spined sticklebacks. Taken together, our analyses of substitution rates, positive selection and gene loss suggest that there are considerable genetic differences between these two ecologically and phenotypically similar species.

### Genetic divergence between marine and freshwater nine-spined sticklebacks

Much research has been directed towards investigating genome-wide divergence between marine and freshwater three-spined sticklebacks and many genes associated with their divergence have been identified [3, 68]. Genetic differentiation between marine and freshwater nine-spined sticklebacks also has been described in studies utilizing microsatellites [41] and restriction-site-associated DNA sequencing [42]. For example, Shikano et al. [41] found several functionally- and physiologically-important genes that had experienced divergent selection between different habitats, and Bruneaux et al. [42] showed that genomic regions enriched for genes having functions related to immunity, chemical stimulus response, lipid metabolism, and signaling pathways had experienced positive selection. However, in-depth genome-wide studies of genetic differentiation between marine and freshwater nine-spined sticklebacks have been lacking. Here, we probed the genome-wide genetic differentiation between marine and freshwater nine-spined sticklebacks to understand whether similar or different genetic changes underlying divergence between freshwater and marine populations exist in the two stickleback species. We found 25 genes with ‘fixed’ genotypes between marine and freshwater nine-spined sticklebacks (Additional file 3: Table S8), and these represent candidates for ecotypic differentiation in nine-spined sticklebacks. Interestingly, one of these genes, the *enolase 1a* (ENSGACG00000007396) gene has also been found to be associated with the divergence of marine and freshwater three-spined sticklebacks [3]. *ATPases* are another group of interesting genes that have been associated with the marine and freshwater divergence in sticklebacks. We found that the *ATP5B* and *ATP6v1ba* genes have SNPs differentiating marine and freshwater nine-spined sticklebacks, and similar evidence is available from *ATP6V1Aa*[41] in nine-spined and *ATP6V0A1* and *ATP6V0E1* in three-spined sticklebacks [3]. Furthermore, a transferrin gene (ENSGACG00000013533) with a putative function in iron ion transport may be of particular interest for understanding adaptive population divergence of marine and freshwater nine-spined sticklebacks, since ion concentration is one of the notable environmental differences demarcating marine and freshwater habitats. Hence, *enolase 1a*, *ATP5B*, *ATP6v1ba* and transferrin provide promising candidates for further investigations focused on understanding the molecular mechanisms of differentiation and adaptation between marine and freshwater stickleback populations. Further studies screening more populations and individuals are needed to evaluate the robustness of these results, as well as to understand how often adaptive divergence between marine and freshwater populations of different stickleback taxa is occurring through evolution in the same or in different genes or genetic elements.

## Conclusions

With the massively parallel pryrosequencing of nine-spined stickleback cDNA libraries, we identified over three thousand unique gene transcripts and hundreds of genic microsatellites. Using these transcripts, we calculated sequence substitution rates in coding regions, in UTRs, and across synonymous sites between nine- and three-spined sticklebacks. We identified over a hundred genes with molecular patterns of positive selection in one or both stickleback lineages and found several candidate genes that might be involved in differential adaptation between marine and freshwater nine-spined sticklebacks. Both the same and different genes were found to associate with marine and freshwater divergence across stickleback taxa. Apart from these specific findings, the study brings about significant amount of new resources (*viz*. gene sequences, microsatellites, and SNPs) to the reach of the research community interested in fish and stickleback genomics in particular.

## Methods

This study did not involve human subjects, and our experimental protocol was approved by the ethics committee of National Animal Experiment Board, Finland (permission numbers: ESLH-STSTH223A and STH037A).

### Fish sampling, RNA isolation, and cDNA library construction

We sampled two male and two female nine-spined sticklebacks from the Baltic Sea (Helsinki, Finland; 60°12′N, 25°11′E), and one male and one female from an isolated freshwater pond (Rytilampi, Finland; 66°23′N, 19°19′E). We chose to sequence the brain and liver transcriptomes to access a large number of diverse transcripts, as these are highly complex organs with complex transcriptomes. Total RNA was extracted from brain and liver tissues using TRIzol reagent (Invitrogen, Carlsbad, CA, USA) according to the manufacturer’s protocol. We constructed four cDNA libraries (marine brain, marine liver, freshwater brain, and freshwater liver) with the SuperScript® Double-Stranded cDNA Synthesis Kit (Invitrogen, cat. no. 11917-010), according to the manufacturer’s protocol. Equimolar amounts of the total RNA from each of the two males and two females from marine population were pooled for construction of the marine brain library, but only one male and one female were used for the marine liver library. Likewise, RNA from one male and one female were used for the freshwater brain and liver libraries.

### Transcriptome sequencing and assembly

We barcoded the four cDNA libraries and sequenced them in a half plate of GS FLX (Roche 454) Standard Chemistry run by DNA Sequencing and Genomics Laboratory, Institute of Biotechnology, University of Helsinki at Helsinki, Finland. Sequences have been deposited in the NCBI Short Read Archive (Accession no. SRR846896, SRR846899, SRR846900, and SRR846901). We trimmed adaptors and low quality reads using custom Perl scripts. We then assembled the cleaned reads using v2.5.3 of the GS *De Novo* Assembler [69] into contigs representing four transcriptomic libraries, one brain and one liver library from each population. We obtained three additional transcriptomic libraries by pooling the contigs from the four cDNA libraries into a marine transcriptome, a freshwater transcriptome and the all-encompassing ‘nine-spined stickleback transcriptome’. These transcriptomic libraries were assembled from reads using a minimum overlap length of 40 bp and a minimum overlap identity of 98%. Detailed information of the transcriptome assemblies are listed in Additional file 2: Table S1.

### Gene annotation

Our annotations focused on the ‘nine-spined stickleback transcriptome’ that was assembled with all reads combined from the four cDNA libraries. We only included assembly contigs with a minimum length of 100 bp for further analyses and used two comprehensive methods to annotate the remaining contigs. We first assigned their putative identities using BLASTX [70] searches against protein datasets of the three-spined stickleback reference (a freshwater individual [3]) from the Ensembl database [71] (release-68) with an E-value cutoff of 1 × 10^-10^, and paired the contigs with their top BLAST hit. The resulting gene pairs are herein referred to as orthologs. Importantly, because of varying transcript lengths and alternative transcription, different nine-spined stickleback contigs can map to different regions or to alternative transcripts of the same three-spined stickleback gene. To identify genes that are possibly lost (or missing) from the three-spined stickleback genome, we used contigs without hits against three-spined stickleback proteins as queries in BLASTX searches against protein datasets of the other model fishes *Danio rerio*, *Gadus morhua*, *Oreochromis niloticus*, *Oryzias latipes*, *Takifugu rubripes*, and *Tetraodon nigroviridis* from the Ensembl database release-68 and *Xiphophorus maculatus* from the Ensembl database release-70. We then used those contigs with hits in other model fish as queries in BLASTN and BLAT [72] searches against the three-spined stickleback genome to validate that these putative genes are lost (or missing) from the three-spined stickleback genome.

We assigned putative functions for each selected nine-spined stickleback contig using version 2.5.0 of Blast2GO [73], which performs a BLASTX search against the non-redundant database from NCBI with default parameters. We obtained annotated accession numbers and Gene Ontology (GO) [74] numbers from NCBI QBLAST [70] based on an E-value of 1 × 10^-10^ and a high-scoring segment pair cut-off greater than 33. We conducted the annotation procedure with the following parameters: a pre-E-value-Hit-Filter of 10^-6^, a pro-Similarity-Hit-Filter of 15, an annotation cut-off of 55, and a GO weight of 5. GO term enrichment test was conducted using GOSSIP [75].

To obtain putative protein-coding and amino acid sequences, we employed GeneWise2 [76] to deduce the open reading frame for each contig sequence using its corresponding best-match protein in the three-spined stickleback as a guide. The putative untranslated region (UTR) of each contig was obtained based on the results of the ORF prediction and further assessed by alignment with UTRs of their corresponding putative orthologs using MUSCLE [77] with default settings to avoid including assembly artifacts.

### Substitution rate estimation

We aligned the amino acid sequences of each pair of orthologs from nine- and three-spined sticklebacks using MUSCLE [77] with default settings and manually inspected for possible alignment artifacts. We performed DNA sequence alignments from the resulting protein alignments using a custom Perl script. The number of nonsynonymous substitutions per nonsynonymous site (*K*_*a*_) and synonymous substitutions per synonymous site (*K*_*s*_) between each orthologous pair was computed using a maximum-likelihood method [78] with the YN00 program implemented in PAML version 4.4 [79]. Only nine-spined stickleback contigs with *K*_*s*_ ≤ 0.5 compared to their three-spined stickleback orthologs were selected for further analyses (e.g., GO annotation and SNP calling) and are referred to as unigenes. In addition, if different nine-spined stickleback contigs aligned to the same three-spined gene, nine-spined stickleback contig with smallest *K*_*s*_ to the three-spined gene was kept; in case two contigs aligned to the same three-spined gene with equal *K*_*s*_ values, we randomly kept one of them for further analysis. As mentioned previously, several nine-spined stickleback contigs or unigenes can correspond to different regions or transcripts of the same three-spined stickleback gene.

We estimated the overall substitution rate between the nine- and three-spined stickleback genomes based on the divergence between unigenes and their orthologs (coding region and UTRs at least 50 bp long) while considering a divergence time around 13 Mya [22]. Distances of coding regions and UTRs were calculated separately using Kimura’s two parameter (K2P) model [80] in EMBOSS [81].

We performed the branch-site test [82, 83] with the codeml program in PAML [79] to detect positive selection operating on sites in the nine- and three-spined stickleback lineages. For this test, we used the corresponding 1-to-1 ortholog in *O. latipes* (determined from Ensembl) as an outgroup. We were able to perform this test for 2,458 unigenes. We calculated the *P* values based on the Chi-square critical values of 3.84 (5%) as recommended in PAML [79]. Multiple test correction was performed using the qvalue package in R [84] with default settings to correct for the false discovery rate (FDR).

### SNP calling

To determine single nucleotide polymorphisms (SNPs) among sampled nine-spined stickleback individuals, we mapped all of the cleaned reads from each of the four cDNA libraries separately to the nine-spined stickleback unigenes using BWA-SW in BWA 0.6.1 [85] with default settings. SNPs from each of the four mappings were called using samtools 0.1.18 [86] with mpileup -I to disable indel calling as insufficient flushing during 454 sequencing usually leads to indel events around homopolymers [87]. Only bases with a Phred quality score of at least 20 were considered for the SNP calling. Combined with the three-spined stickleback ortholog, SNPs were used for performing the McDonald-Kreitman (MK) test of neutral evolution [88] using libsequence [89]. The MK test is used for evaluating the ratio of polymorphism (intraspecies differences) and divergence (interspecies differences) at nonsynonymous and synonymous sites. Under neutrality, the ratio of polymorphism and divergence at these site classes is equal. We calculated an unbiased estimator of the direction of selection (DoS) developed by Stoletzki & Eyre-Walker [90], which is a modification of the neutrality index (NI) [91] by calculating the difference between the proportion of divergent and polymorphic nonsynonymous substitutions. Whereas DoS is zero under neutrality, positive selection driving an excess of nonsynonymous divergence between species would render DoS positive, and purifying selection reflected by an excess of nonsynonymous polymorphisms within species would decrease DoS below zero. Statistical significance in the departure from neutrality for each gene was determined by the Chi-square test with Yates correction as implemented in libsequence [89].

### Microsatellite identification

We used a microsatellite identification program – MISA [92] to identify microsatellite motifs in our nine-spined unigenes. We searched for all types of Simple Sequence Repeats (SSRs) from mononucleotide to hexanucleotides using the following parameters: at least 10 repeats for mono-, 6 repeats for di- and 5 repeats for tri-, tetra-, penta- and hexanucleotide for simple repeats. We identified both perfect (SSRs containing a single repeat motif) and compound (SSRs composed of two or more motifs separated by < 100 bp) SSRs.

## Availability of supporting data

The data sets supporting the results of this article are available in the in the National Center for Biotechnology Information Short Read Archive repository (Accession no. SRR846896, SRR846899, SRR846900, and SRR846901), and included within the article and its additional files.

## Electronic supplementary material

Additional file 1: Figure S1: Read length distribution; **Figure S2.** Contig length distribution; **Figure S3.** Unigene GO annotation plotting with WEGO; **Figure S4.** Unigene length distribution; **Figure S5.** Chromosomal distribution of unigenes along the three-spined stickleback genome (grey) and genes with molecular patterns of adaptive evolution between nine- and three-spined sticklebacks (black); **Figure S6.** Gene-specific substitution rates per synonymous site per year between nine- and three-spined sticklebacks; **Figure S7.** Boxplot of pairwise *K*_*a*_ and *K*_*s*_ between nine- and three-spined sticklebacks of the 165 genes showing evidence for adaptive evolution and other genes. (DOCX 3 MB)

Additional file 2: Table S1: Assembly summary of nine-spined stickleback transcriptomic libraries; **Table S2.** Genes putatively lost in three-spined sticklebacks. (DOCX 31 KB)

Additional file 3: Table S3: Results from pairwise Ka/Ks calculation for detection of positive selection between nine- (9SS) and three-spined (3SS) sticklebacks; **Table S4.** Results from a branch-site test for positive selection; **Table S5.** Direction of Selection (DoS) from the MK test; **Table S6.** GO term enrichment test of the genes with adaptive evolution between nine- and three-spined sticklebacks using GOSSIP; **Table S7.** Genes with SNPs between ecotypes; **Table S8.** Fixed genotype differences between ecotypes; **Table S9.** SSRs in unigenes of nine-spined stickleback. (XLSX 116 KB)
