# Supplementary material for: Genomic divergence between nine- and three-spined sticklebacks
Source: BMC Genomics. 2013 Nov 5;14(1):756. doi: 10.1186/1471-2164-14-756 (PMC4046692; doi:10.1186/1471-2164-14-756)

Supp_Fig.1 Read length distribution. Read number of each nine-spined stickleback transcriptomic library is given. HKI_B: the marine brain library (Helsinki); HKI_L: the marine liver library (Helsinki); RYTI_B: the freshwater brain library (Rytilampi); RYTI_L: the freshwater liver library (Rytilampi).


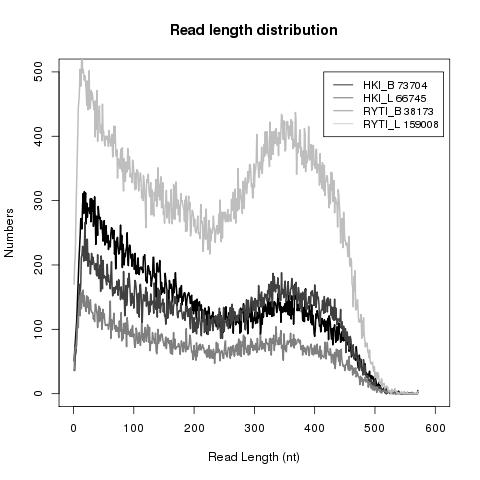


Supp_Fig.2 Contig length distribution (of 7,932 contigs > 100bp).


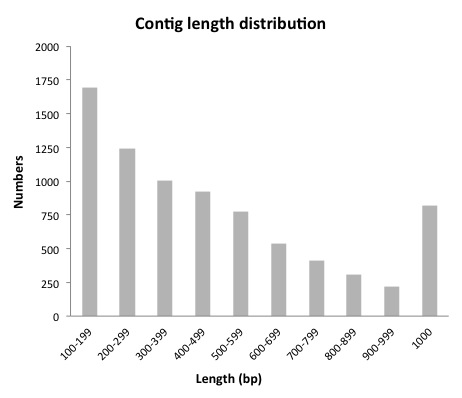


Supp_Fig.3 “Unigene” GO annotation plotting with WEGO.


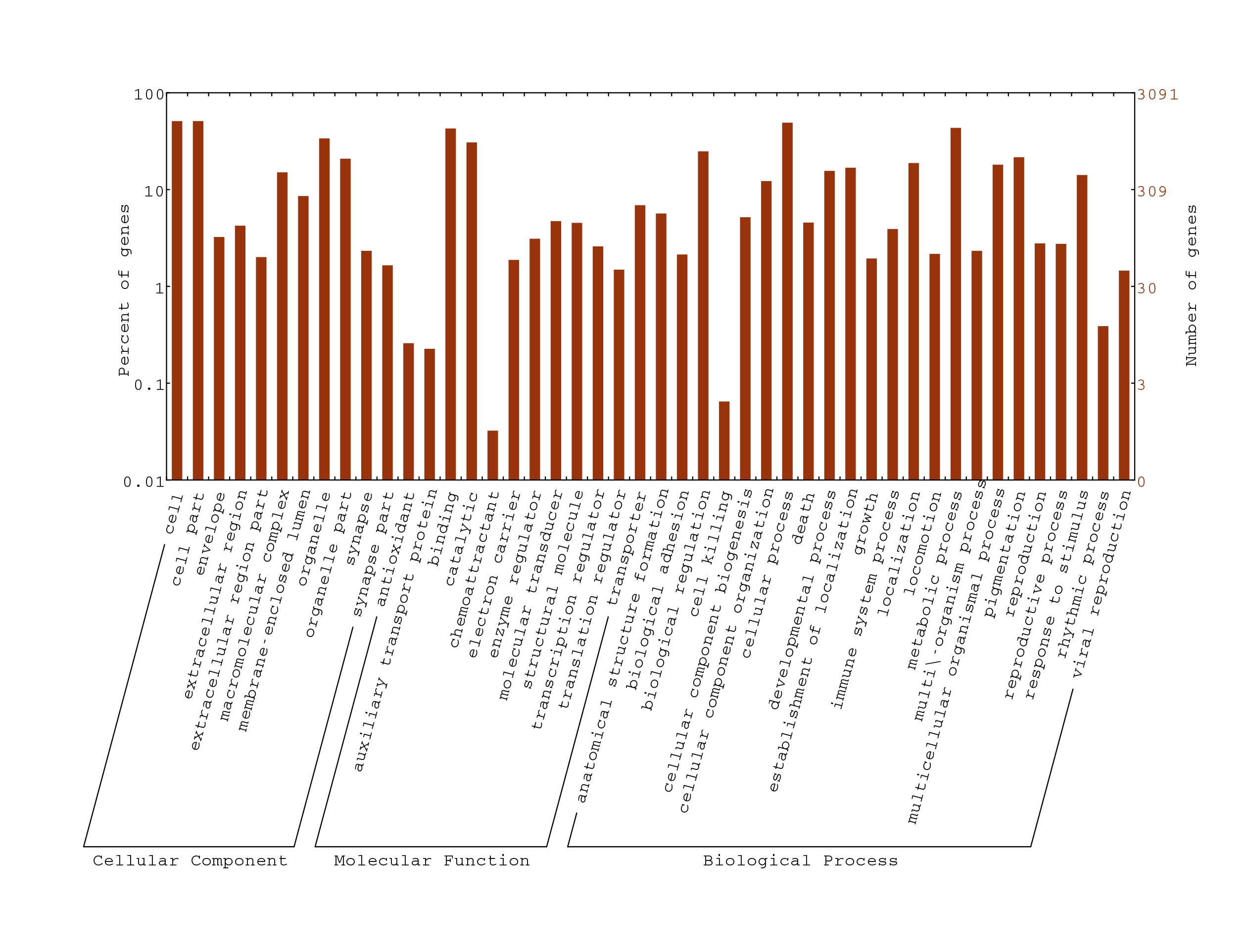


Supp_Fig.4 “Unigene” length distribution.


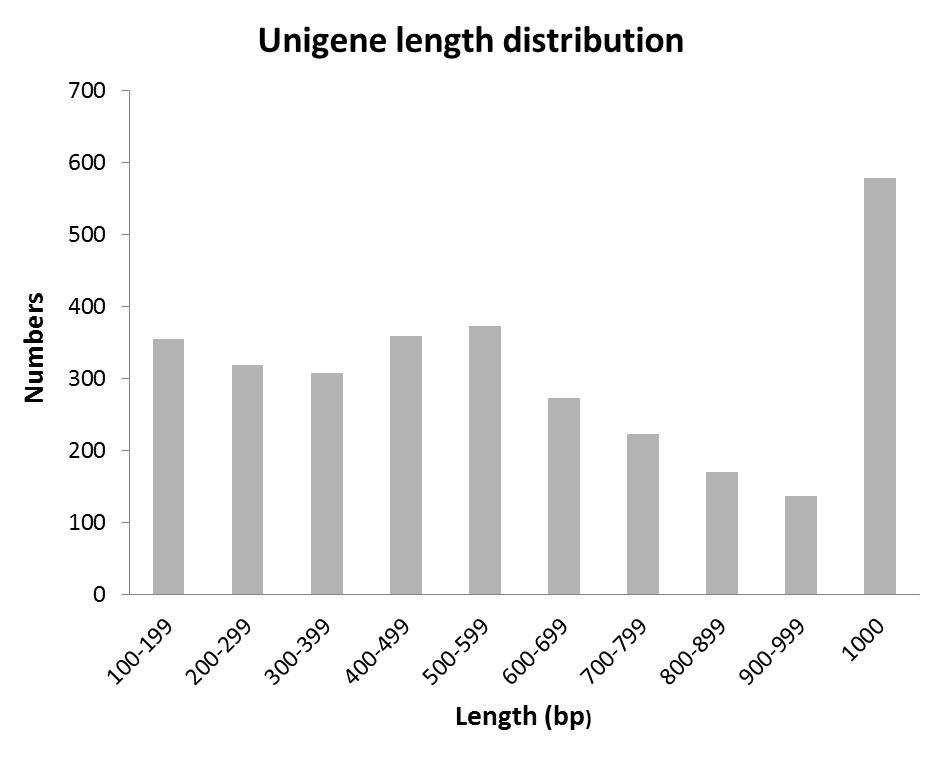


Supp_Fig.5 Chromosomal distribution of “unigenes” along the three-spined stickleback genome (grey) and genes with molecular patterns of adaptive evolution between nine- and three-spined sticklebacks (black).


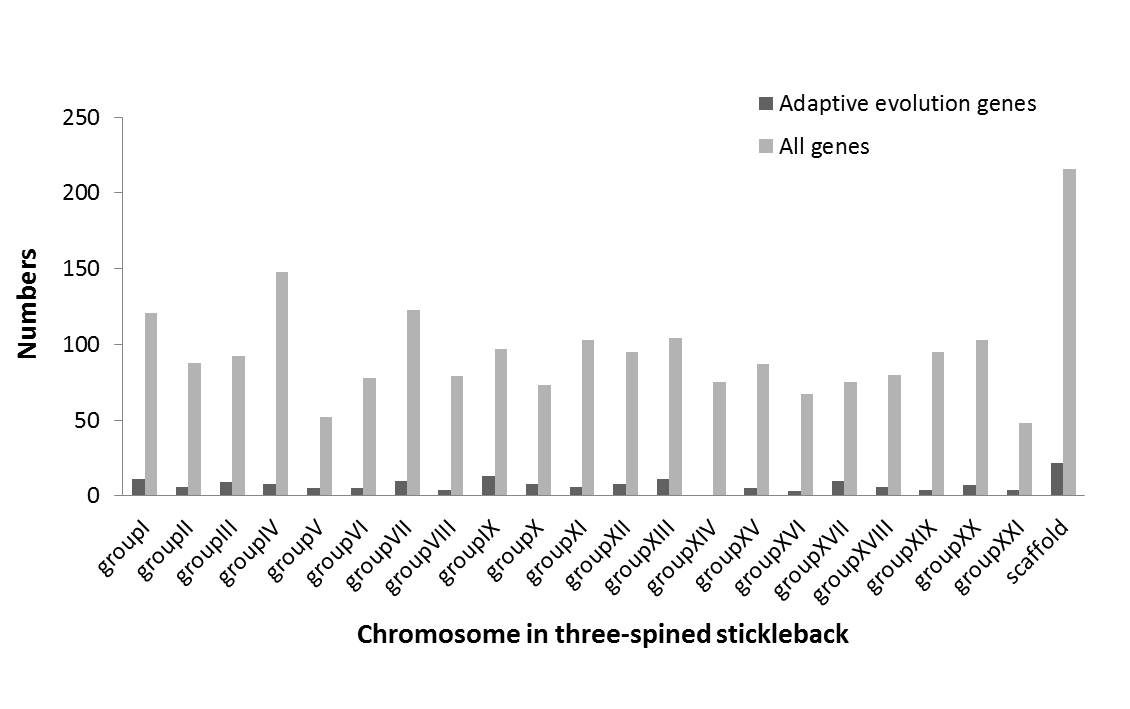


Supp_Fig.6 Gene-specific substitution rates per synonymous site per year between nine- and three-spined sticklebacks.


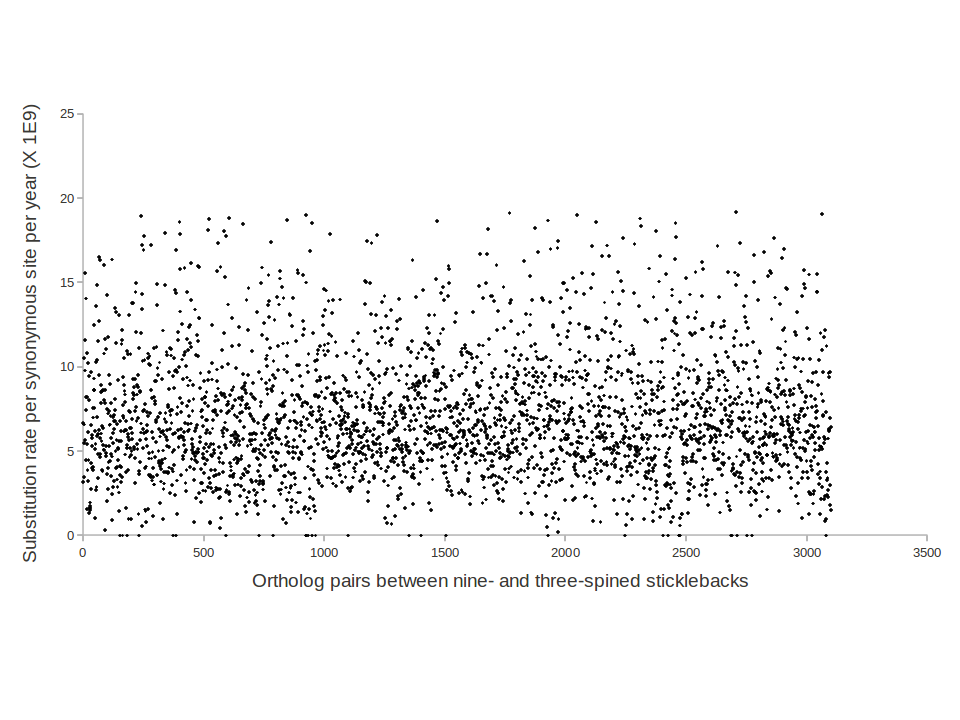


Supp_Fig.7 Boxplot of pairwise *K_a_* and *K_s_* between nine- and three-spined sticklebacks of the 165 genes showing evidence for adaptive evolution and other genes.


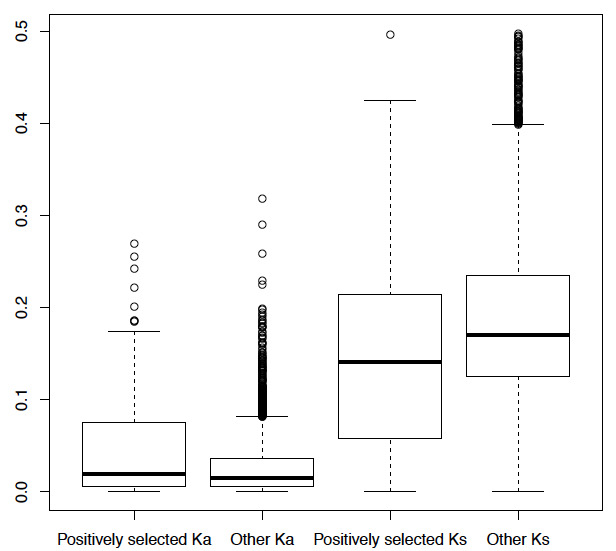

Supplement: Supplementary file 1 — Additional file 1: Figure S1: Read length distribution; Figure S2. Contig length distribution; Figure S3. Unigene GO annotation plotting with WEGO; Figure S4. Unigene length distribution; Figure S5. Chromosomal distribution of unigenes along the three-spined stickleback genome (grey) and genes with molecular patterns of adaptive evolution between nine- and three-spined sticklebacks (black); Figure S6. Gene-specific substitution rates per synonymous site per year between nine- and three-spined sticklebacks; Figure S7. Boxplot of pairwise K a and K s between nine- and three-spined sticklebacks of the 165 genes showing evidence for adaptive evolution and other genes. (DOCX 3 MB) [file 12864_2013_5474_MOESM1_ESM.docx]
