# Supplementary material for: Genomic divergence between nine- and three-spined sticklebacks
Source: BMC Genomics. 2013 Nov 5;14(1):756. doi: 10.1186/1471-2164-14-756 (PMC4046692; doi:10.1186/1471-2164-14-756)
Supplement: Supplementary file 2 — Additional file 2: Table S1: Assembly summary of nine-spined stickleback transcriptomic libraries; Table S2. Genes putatively lost in three-spined sticklebacks. (DOCX 31 KB) [file 12864_2013_5474_MOESM2_ESM.docx]

Table 1 Assembly summary of nine-spined stickleback transcriptomic libraries

| Assembly series | MOL (bp) | MOI (%) | Total and assembled read | Contig ≥ 100 bp | | |
| --- | --- | --- | --- | --- | --- | --- |
|  |  |  |  | **Total number** | **Average length** | **Median length** |
| HKI_BL_RYTI | 40 | 98 | 337,630/181,242 | 7,932 | 520 | 403 |
| HKI_B | 40 | 100 | 73,704/16,087 | 1,024 | 337 | 290 |
| HKI_L | 40 | 100 | 66,745/28,389 | 865 | 408 | 340 |
| HKI_BL | 40 | 100 | 140,449/47,630 | 2,031 | 370 | 310 |
| RYTI_B | 40 | 100 | 38,173/5,986 | 431 | 377 | 322 |
| RYTI_L | 40 | 100 | 159,008/63,309 | 2,404 | 461 | 382 |
| RYTI_BL | 40 | 100 | 197,181/72,713 | 2,946 | 455 | 382 |

HKI_B: the marine brain library (Helsinki); HKI_L: the marine liver library (Helsinki); RYTI_B: the freshwater brain library (Rytilampi); RYTI_L: the freshwater liver library (Rytilampi); HKI_BL_RYTI: the 4 libraries combined; MOL: minimum overlap length; MOI: minimum overlap identity.

Table 2 Genes putatively lost in three-spined sticklebacks

| Contig in nine-spined stickleback | | Hit gene name | Hits in other teleosts | | | | | | |
| --- | --- | --- | --- | --- | --- | --- | --- | --- | --- |
| Name | **Length (bp)** |  | ***D. rerio*** | ***G. morhua*** | ***Ore. niloticus*** | ***Ory. Latipes*** | ***Ta. rubripes*** | ***Te. nigroviridis*** | ***X. maculatus*** |
| contig02920 | 140 | SLC20A2 | - | - | - | ENSORLP00000002277* | - | - | - |
| contig03059 | 801 | THAP9 | ENSDARP00000071844 | ENSGMOP00000018629 | ENSONIP00000013799 | - | - | - | ENSXMAP00000001605 |
| contig03533 | 189 | LAMC1 | - | - | ENSONIP00000000384 | - | - | - | - |
| contig03535 | 145 | LAMC1 | - | - | - | - | ENSTRUP00000034302 | ENSTNIP00000004862 | - |
| contig03702 | 322 | Kidins220 | ENSDARP00000092376 | - | ENSONIP00000023703 | ENSORLP00000022721 | ENSTRUP00000013168 | ENSTNIP00000007449 | ENSXMAP00000009750 |
| contig03703 | 233 | Kidins220 | ENSDARP00000092376 | - | ENSONIP00000023703 | ENSORLP00000022721 | ENSTRUP00000013168 | ENSTNIP00000007449 | ENSXMAP00000009750 |
| contig04059 | 202 | HYDIN | ENSDARP00000119696 | - | ENSONIP00000016629 | - | - | - | ENSXMAP00000015205 |
| contig04181 | 205 | LAMA1 | - | - | ENSONIP00000019013 | - | ENSTRUP00000007821 | ENSTNIP00000016516 | ENSXMAP00000012100 |
| contig04182 | 223 | LAMA1 | - | - | ENSONIP00000019013 | - | ENSTRUP00000007822 | ENSTNIP00000004687 | ENSXMAP00000012100 |
| contig04183 | 172 | LAMA1 | - | - | ENSONIP00000019013 | - | ENSTRUP00000007821 | - | ENSXMAP00000012100 |
| contig04193 | 387 | C7orf57 | - | - | - | ENSORLP00000002310 | - | - | ENSXMAP00000002172 |
| contig04194 | 152 | C7orf57 | - | - | - | - | - | - | ENSXMAP00000002172 |
| contig06259 | 774 | SDHD | ENSDARP00000072578 | - | ENSONIP00000018565 | ENSORLP00000012700 | ENSTRUP00000031467 | ENSTNIP00000000936 | ENSXMAP00000008310 |
| contig06985 | 585 | C5 | ENSDARP00000088095 | - | - | ENSORLP00000021960 | ENSTRUP00000032286 | ENSTNIP00000008583 | ENSXMAP00000004243 |
| contig08243 | 374 | ERBB4 | - | - | ENSONIP00000016825 | ENSORLP00000002975 | ENSTRUP00000037399 | ENSTNIP00000011153 | ENSXMAP00000007916 |

*: The underlined IDs are the best blast hits with nine-spined stickleback contigs.
